# Optimizing Bacteriophage Screening and Isolation Methods for Microbial Samples Derived from Different Body Sites of Cattle

**DOI:** 10.3390/microorganisms14061385

**Published:** 2026-06-22

**Authors:** Gabriela Magossi, Godson Aryee, Samat Amat

**Affiliations:** Department of Animal Sciences, North Dakota State University, Fargo, ND 58105, USA

**Keywords:** bacteriophages, phage-screening methods, antibiotic alternative, commensals, bovine pathogens, cattle, whole-genome sequencing

## Abstract

Bacteriophages are increasingly investigated as tools for studying and manipulating microbial communities in cattle. However, phage isolation remains challenging because of host specificity, microbial ecosystem differences, and the lack of optimized screening approaches. The objectives of this study were to (i) optimize the phage-screening method for microbial samples obtained from different cattle body sites, (ii) isolate lytic phages against key bovine pathogens and commensal bacteria, and (iii) characterize the isolated phages and their bacterial hosts. A total of 1214 samples from different cattle body sites (*n* = 1194) and environmental sources (*n* = 20) were screened using 13 phage detection methods, including one high-throughput approach. Eighty-three phages were isolated, primarily from ruminal fluid (59), feces (15), vaginal (7) and nasopharyngeal swabs (1), and fetal ruminal fluid (1). The bacterial hosts inhibited by these phages were from 29 genera, with *Bacillus* (34), *Escherichia*/*Shigella* (8), *Shouchella* (5), *Corynebacterium* (4), and *Lysinibacillus* (4) being the most common. No phages were identified against bovine pathogens including *Trueperella pyogenes*, *Mannheimia haemolytica*, *Pasteurella multocida*, or *Moraxella bovis.* Method 12 demonstrated the highest efficiency in phage recovery, particularly from ruminal samples. The successful recovery of bacteriophages from gastrointestinal, reproductive, respiratory, and fetal bovine samples demonstrates the utility of the optimized screening methods for isolating phages from diverse cattle-associated microbial ecosystems. Further studies are needed to refine these approaches to improve the recovery of phages targeting bovine pathogens.

## 1. Introduction

Bacteriophage screening against bovine bacterial pathogens is essential due to the rising antimicrobial resistance (AMR) in pathogens associated with bovine respiratory disease (BRD) [1], liver abscesses [2], mastitis [3] and bovine infectious keratoconjunctivitis (pinkeye) [4]. The increasing prevalence of multidrug resistant pathogens in cattle poses significant threat to the human, animal and environmental health, highlighting the need for developing alternatives to antibiotics to maintain sustainable livestock production globally. Bacteriophage therapy offers a promising alternative to antibiotics [5]. Bacteriophages are viruses that specifically infect and lyse bacterial cells [6,7]. Based on life cycle, phages are categorized into lytic and lysogenic (temperate) phages, with the former being the ideal for the phage therapy due to its capacity of rapid distribution and destruction of host bacterial cells [8].

Phage therapy has been evaluated for treating or preventing infectious diseases in farm animals. For example, bacteriophages have been tested as prophylaxis and treatment for enteropathogenic *E. coli* in calves [9], piglets [10], and small ruminants [11], and showed promising efficacy in reducing mortality and morbidity in treated animals [12]. The phages shed in the feces of those phage cocktail-receiving animals also showed improvement in animal health after being used for fecal transplantation [13]. The efficacy of phage cocktails against seven enteropathogenic *E. coli* strains in preventing and treating diarrhea in calves has also been previously demonstrated [13]. Phage therapy has also been successfully employed to control *Campylobacter jejuni* and *Campylobacter coli* [14], and to reduce mortality in chickens inoculated with pathogen *Salmonella typhimurium* [15].

In addition to the application of phage therapy as an antimicrobial alternative to control infectious diseases in food-producing animals, recently phages have garnered increasing research interest due to their potential use in precise modulation of microbiome and host–microbiome interactions [16,17]. Phages are highly specific to their bacterial hosts and thus have minimal to no effect on the other commensal members of the microbiome where the phage host resides [18]. Cattle body harbors diverse, site-specific and complex microbial communities. Microbiomes associated with gastrointestinal, reproductive and respiratory tracts, as well as oculus and mammary glands are important in maintaining overall cattle health and productivity [19,20]. Among the different microbiomes present in and on cattle body, the ruminal microbiome plays an important role in defining feed efficiency, enteric methane emissions, and overall host health and immune development [21,22]. The reproductive microbiomes (male and female) have also been increasingly known for their importance in defining reproductive health and fertility [23,24,25,26]. In addition, the respiratory microbiome is also important in maintaining pulmonary health via providing colonization resistance against BRD pathogens [27,28,29,30]. Thus, developing bacteriophage-based microbiome manipulation to improve feed efficiency, enteric methane mitigation, and gut, respiratory and reproductive health in cattle [31] holds a great potential in such an era where cattle production continues under pressure to improve its output while reducing the environmental impact and reliance on antibiotic usage [32].

Sampling nature, bacterial cell density, and microbiome complexity differ across the gut, reproductive and respiratory tracts in cattle. This coupled with the potential bacterial cell exchanges between different microbial ecosystems (gut, respiratory and reproductive tracts) within a cattle body highlights the necessity to optimize a bacteriophage-screening method that can be used for screening different sample types with an optimal phage recovery rate. Isolating and characterizing phages from different bovine tissues, fluids, and swabs come with several challenges. These challenges could be overcome by refining the process involved with sampling, phage enrichment, and plaque assays. Classical methods of screening and isolating bacteriophages was developed in the early 20th Century [33], and the current most common method used for bacteriophage screening from environmental samples is the double-layer agar assay [34,35,36,37,38]. The double-layer agar method has been modified and improved over the years [33,39]. Double-layer agar-based phage screening has been applied to ruminal samples with a limited phage isolation success rate [40], despite the belief that the rumen harbors a relatively large phage population (10^9^ and 10^11^ viral particles/mL of ruminal fluid) [41] comprising more than 25 lytic morphotypes [40,42,43]. Lytic phages targeting specific ruminal bacteria such as *Streptococcus bovis* [44,45,46], *Bacteroides ruminicola* [47], *Ruminococcus* [48], and *Butyrivibrio fibrosolvens* [49] have been isolated. Despite the limited number of phages having been isolated from ruminal ecosystem, the recent metagenomic data [43] suggest significant potential for new phage discoveries within the rumen that could be used to manipulate ruminal microbiome composition, its functions and ruminal fermentation.

Thus, there is pressing need to optimize the current available bacteriophage methods for screening microbial samples originating from different cattle anatomical sites. Including but not limited to the rumen, respiratory, and reproductive tracts, and fetal-associated microbial samples. In the present study, we screened a relatively large number of microbial samples originating from different cattle body sites for isolation of phages that can inhibit bovine pathogenic bacteria and commensal bacterial members. We optimized phage-screening methods that could be used for phage screening from microbial samples obtained from different cattle body sites. In addition, we performed whole-genome sequencing and microscopic imaging on a subset of isolated phages to gain genomic and morphological insights into those bovine origin bacteriophages.

## 2. Materials and Methods

### 2.1. Overview of Animal Studies and Sample Sources for Bacteriophage Screening

The samples used for bacteriophage screening in this study originated from seven beef cattle studies conducted at North Dakota State University (NDSU), Fargo, ND, USA. Across these studies, more than 1000 samples were collected from multiple anatomical sites, including the gastrointestinal, respiratory, reproductive, ocular, and fetal compartments. All experimental procedures were approved by the NDSU Institutional Animal Care and Use Committee (IACUC). The studies included dietary intervention trials (Trials 1–4 and 7), microbiome characterization studies (Trials 5 and 6), and pregnancy/developmental programming studies (Trials 3, 4, and 7).

Animal Trial #1 (hempseed cake study; IACUC protocol A21010) evaluated the effects of feeding 20% hempseed cake compared with dried distillers’ grains with solubles (DDGS) on the gastrointestinal, respiratory, and reproductive microbiota of Angus-crossbred heifers housed at the NDSU Beef Cattle Research Complex (BCRC) [50].

Animal Trial #2 (Lactipro study; IACUC protocol 20210068) evaluated the impact of supplementing Lactipro NXT *Megasphaera elsdenii* probiotic to yearling beef heifers fed a high-energy diet at the NDSU BCRC. Ruminal fluid samples (*n* = 81) from this study were included in the present phage-screening analysis.

Animal Trial #3 (AFRIMMAT study; IACUC protocol 20210043) investigated the effects of maternal dietary modulation using high-forage or high-concentrate diets during gestation on offspring microbiome development, animal performance, and enteric methane emissions. Samples used for phage screening included ruminal fluid (*n* = 89), vaginal swabs (*n* = 11), nasopharyngeal swabs (*n* = 14), and fecal samples (*n* = 4).

Animal Trial #4 (one-carbon metabolite study; IACUC protocol A21049) evaluated the effects of one-carbon metabolite supplementation during gestation on fetal and offspring development [51]. Pregnant beef heifers received dietary treatments containing methionine, choline, folate, and vitamin B12 and were maintained until approximately 260 days of gestation. Samples used for phage screening included ruminal fluid (*n* = 79), vaginal swabs (*n* = 78), nasopharyngeal swabs (*n* = 32), fecal samples (*n* = 22), fetal ruminal fluid (*n* = 20), and fetal meconium samples (*n* = 19).

Animal Trial #5 (pregnancy efficiency study; IACUC protocol A21061) characterized vaginal and uterine microbiota associated with pregnancy establishment in beef cattle [26]. Vaginal and uterine swabs collected from cows and heifers at the time of artificial insemination were used for phage screening in the present study.

Animal Trial #6 (pinkeye study; IACUC protocol 20220029) characterized the ocular microbiota associated with infectious bovine keratoconjunctivitis (pinkeye) in beef cattle. Ocular swabs were collected from cattle with and without pinkeye originating from both NDSU research herds and commercial cow–calf operations in North Dakota, USA.

Animal Trial #7 (vitamin and mineral supplementation study; IACUC protocol A21047) evaluated the multigenerational effects of maternal vitamin and mineral supplementation during gestation on fetal and offspring development [52]. Ruminal fluid and vaginal swabs collected from pregnant heifers were included in the present phage-screening study.

Except for Trial #6, cattle used across the studies originated primarily from the same NDSU Central Grasslands Research Extension Center (CGREC) cow–calf herd, thereby reducing variability associated with genetic background, herd management, and environmental conditions. Animal management and sample collection procedures across studies were performed by the same research personnel using standardized protocols. Information regarding antimicrobial administration in the individual studies is provided in the corresponding original publications when applicable.

### 2.2. Sample Collection for Phage Screening

#### 2.2.1. Ruminal Fluid (RF) Sample Collection

Ruminal samples were collected from Angus cattle and these samples were sampled from beef steers, dams and heifers, as well as dairy cattle. The cattle were restrained in a hydraulic cattle chute and a metal speculum was placed in the mouth so that a flexible PVC tube could be passed through the esophagus and into the rumen as described in our previous publications [50,51]. The tube was worked through the ruminal mat and then a light vacuum was applied to retrieve the ruminal fluid. The ruminal fluid was collected into a clean plastic side-arm Erlenmeyer flask, gently swirled, and aliquoted into either a sterile 15 or 50 mL falcon tubes. Tubes containing ruminal fluid used for phage screening were stored in warm water, transported to the lab and processed upon arrival.

#### 2.2.2. Vaginal Swab (VS) Sample Collection

The vaginal swab samples were collected from both dams and heifers [26,50,51]. While animals were restrained in a mechanical chute, one or two sterile cotton-tipped swabs (10 cm) were inserted simultaneously into the vagina and vaginal canal to collect as much mucous material as possible. The swab tips were broken and placed into 2 mL cryovials. Then, a combination of up to 12 swabs, originating from 6 different animals, were combined in 50 mL centrifuge falcon tubes and kept on ice and transported to the lab.

#### 2.2.3. Uterine Swab (US) Sample Collection

The uterine swab samples were collected from Angus-bred cows, as described previously [26,50,51]. Briefly, double-guarded culture swabs (Reproduction Provisions L.L.C., Walworth, WI, USA) were inserted into the uterus, passing the cervix with the guide through rectal palpation. Swab tips were cut and placed into sterile 2 mL centrifuge tubes and kept on ice and transported to the lab.

#### 2.2.4. Deep Nasopharyngeal Swab (DNS) Sample Collection

The deep nasopharyngeal swab (DNS) samples were collected from cattle as described previously [52]. Briefly, animals were restrained in a mechanical chute, DNSs were inserted into the right nostril of each animal after the outer region of the nostril was cleaned with paper towels. Swab tips were cut with 70% ethanol sterilized metal pliers, placed in sterile 1.5 mL microcentrifuge tubes and stored on ice until brought back to the lab.

#### 2.2.5. Ocular Swab (OS) Sample Collection

Ocular swab samples were collected from beef cattle infected with IBK, or healthy herd mates, across the North Dakota, on the field by instructed personnel and shipped to our lab at NDSU at different time points [53]. Ocular swabs were collected from cattle diagnosed with pinkeye and healthy herd mates using Puritan Opti-Swab with Liquid Amies Collection and Transport System (Puritan, Guilford, ME, USA). For swabbing, cattle were placed in a hydraulic squeeze chute and the head was manually restrained. Before swabbing, the sampled eye was wiped with a clean paper towel sprayed with 70% ethanol to remove any debris. Then, eyelids were opened, and the conjunctival and cornea tissues were gently swabbed. Immediately after sample collection, the tips of the swabs were broken and placed in 1 mL of sterile Amies transport medium and stored on ice for transport to the lab.

#### 2.2.6. Fecal Sample Collection

Feces were collected manually while wearing gloves cleaned with 70% ethanol from the rectum of Angus heifers and deposited into sterile whirl-pak bags. Bags were kept on ice until brought back to the lab. Then, using a sterile disposable spoon, one scoop of four different fecal samples were transferred into a 50 mL tube; next, 45 mL of Dulbecco’s phosphate-buffered saline (DPBS; Corning, Corning, NY, USA) was added and the contents vortexed. Tubes were stored at 4 °C until further processing.

#### 2.2.7. Liver Tissue and Liver Abscess Sample Collection

Liver tissue samples were obtained via liver biopsy at the NDSU ANPC as previously described [54]. Briefly, animals were restrained in a mechanical chute and clippers were used to remove the hair between the 10th and 11th intercostal spaces on the right side of the animal. Then, 3 mL of 2% lidocaine hydrochloride was administered as a local anesthetic and the skin cleaned by scrubbing betadine and alcohol around the incision site. Finally, a Tru-Cut Biopsy Device (Merit Medical, South Jordan, UT, USA) was used to obtain the liver tissue sample that was immediately transferred into a sterile 2 mL cryotube, kept on ice, and transported to the lab.

The liver abscess samples were obtained from three different beef cattle; abscesses were dissected from surrounding liver tissue aseptically after the animal was slaughtered and the liver was removed from the carcass. Abscesses were then placed into sterile whirl-pak bags and shipped on ice to our lab at NDSU where they were processed.

#### 2.2.8. Fetal Ruminal Fluid and Meconium Sample Collection

Fetal samples were collected from beef cattle fetuses (*n* = 20; 180-day-old fetuses) harvested at the time of slaughter as described previously [50]. The uterus was removed and the surface cleaned with 70% ethanol; next, an incision was made with a scalpel, and the fetus removed. Fetuses’ organs were removed with their contents intact; then a 50 cc syringe with an 18-gauge needle was used to extract the fetal ruminal fluid from the rumen (gastric/abomasal part), and a sterile scoop was used to transfer meconium into a sterile 15 mL centrifuge tube. Both samples were kept on ice and transported to the lab.

#### 2.2.9. Milk Sample Collection

Two milk samples were obtained from the NDSU Dairy Farm in Fargo (ND, USA). One sample was taken from the collection tank containing milk from all cows (*n* = 100) milked that day, and the second one was taken directly from the teat of an animal with mastitis. Milk samples were placed in sterile 120 mL plastic specimen collection cups.

#### 2.2.10. Runoff and Drinking Water, and Wastewater Sample Collection

The runoff and drinking water samples were collected from the NDSU BCRC with sterile 120 mL specimen collection cups. The runoff water samples were collected from water that pooled at the lower point at the end of the animal holding pens, from the surface ground. The drinking water was collected from the drinking water tanks located in between 2 pens, from two different tanks. The wastewater samples were collected in the Fargo area. Approximately 100 mL of aliquots were stored in plastic specimen cups and kept on ice and transported to the lab.

#### 2.2.11. Soil Sample Collection

The samples were collected using a soil sample probe tool from the soils of or around the area where beef cattle had been housed at the NDSU Beef Unit facility. The core soil was placed into sterile whirl Pak bags and kept on ice and transported to the lab and processed immediately upon arrival in the lab.

#### 2.2.12. Sample Collection for the High Throughput Assay

Samples (*n* = 109) for the high throughput assay were collected from pregnant beef heifers (approx. 260 days of gestation) at time of slaughter at seven time points (sequential days, four different animals each day) from the same project looking into OCM supplementation (animal trial#4, OCM study). These samples included DNS, OS, VS, tracheal (TR) swabs, and RF. A bone saw was used to cut through the skull of each animal halfway up the nasal cavity, and the DNS were obtained by using cotton-tipped swabs into the exposed nasopharyngeal cavity. The OSs were acquired by using cotton-tipped swabs to swab the surface of the eyes, including the inside of both the upper and lower eyelids. Additionally, cotton-tipped swabs were inserted into the trachea after the organs, including the lungs, were removed from the carcasses, reaching the entirety of the length of the trachea tube. Once the rumen was removed from the carcass, the organ surface was cleaned with 70% ethanol and a sterile scalpel was used to make a small incision into the rumen; then, a 50 mL tube was used to collect RF from its contents. Finally, VSs were obtained as described above. The tips of the swabs were removed and placed into 2 mL cryotubes and kept on ice until transported into the lab for processing. Samples were pooled (all NS together, *n* = 4, all VS together, *n* = 4, etc.) and stored in 2 mL cryotubes containing 30% glycerol at −20 °C.

### 2.3. Bacteriophage Host, Culture Conditions, and Their Origins

The bacterial species selected as target hosts for phage screening included pathogens of interest to cattle. These included the BRD-associated bacterial pathogens *Mannheimia haemolytica*, *Pasteurella multocida*, and *Histophillus somni*, the multifaceted opportunistic pathogen *Trueperella pyogenes*, the IBK-associated pathogens *Moraxella bovis* and *Moraxella bovoculi*, as well as the potential foodborne pathogens *Escherichia coli* and *Salmonella typhimurium* (Table 1). Commensal bacteria isolated from animal samples were also used for phage screening. *T. pyogenes* isolates originated from bovine liver abscesses, *M. haemolytica*, *P. multocida*, and *H. somni* were isolated from DNSs of beef cattle. *M. bovis* and *M. bovoculi* were isolated from ocular swab samples of cattle with IBK that were received by the veterinary diagnostic lab (VDL) at NDSU. The *E. coli* and *S. Typhimurium* isolates were part of our culture collection and were cultured from cattle feces. All bacterial cultures were stored at −80 °C in brain heart infusion (BHI; BD, Franklin Lakes, NJ, USA) + 20% glycerol 2 mL cryotubes. They were all cultured using either tryptic soy agar (BD, Franklin Lakes, NJ, USA) with 5% defibrinated sheep’s blood (TSAb) or BHI agar and/or broth and incubated at 37 °C supplemented with 5% CO_2_ under aerobic condition.

To culture and isolate commensal bacteria from the different cattle samples, 1 mL of pooled samples (RF or swabs) were serially diluted by transferring 50 µL of vortexed liquid sample into 450 µL of DPBS (Dulbecco’s phosphate-buffered saline; Thermo Fisher Scientific, Branchburg, NJ, USA). Next, 100 µL of dilutions 10^−1^ and 10^−2^, for swabs, and 10^−4^ and 10^−5^, for RF, were spread plated on TSAb plates and incubated at 37 °C with 5% CO_2_ overnight. After incubation, the colonies with visibly different morphologies within the plate, and between plates were picked and sub-streaked onto new Columbia blood (CB; BD, Franklin Lakes, NJ, USA) agar plates for isolation using a sterile disposable needle. Plates were then incubated at 37 °C + 5% CO_2_ overnight. The overnight grown isolates were transferred into 1 mL of BHI + 20% glycerol in 2 mL cryotubes and stored at −80 °C.

### 2.4. Methods for Phage Detection from Cattle and Environmental Samples

#### 2.4.1. Sample Filtering

A cheesecloth was used to separate larger particles from the RF; then, RF from individual animals were pooled together (4–8 animals per pool), mixed, transferred into 50 mL centrifuge tubes, and centrifuged at 3900× *g* for 15 min at 4 °C. Next, the supernatant of this first centrifugation was transferred to 2 mL microcentrifuge tubes, and centrifuged at 20,000× *g* for 15 min at 4 °C. Finally, the supernatant was filtered using 0.22 µm syringe filters, and the filtrate was used in the downstream processing.

#### 2.4.2. Swab Sample Processing

Vaginal swab tips stored in microcentrifuge or cryotubes were pooled together (from 4 to 8 swabs per pool) in a sterile 15 mL centrifuge tube containing 10 mL of sterile DPBS and thoroughly vortexed (for minimum 20 s). One mL of swab suspension pools was used for culturing while the remaining supernatant was filtered using 0.22 µm syringe filters and 10 mL syringes; then, 100 µL of the filtrates were transferred into 5 mL of BHI broth containing 100–200 µL of a target bacterial isolate culture or pool of up to 5 isolates cultures and incubated at 37 °C shaking at 220 rpm overnight. This was considered an enrichment step. After incubation, the enrichment tubes were centrifuged at 15,000× *g* for 15 min at 4 °C and the supernatants filtered using 0.22 µm syringe filter.

#### 2.4.3. Polyethylene Glycol (PEG) Precipitation

To readily concentrate phage particles that might be present in large volumes of liquid media, a 20% PEG6000 (*w*/*v*) + 2.5 M sodium chloride (NaCl) solution was added to the liquid at a 5:1 ratio (*v*/*v*) of sample filtrate to the PEG + NaCl solution [55,56]. Then, the mixture was wrapped in aluminum foil and incubated at 4 °C for 48 h. After 48 h, the solution was transferred into 2 mL microcentrifuge tubes, and centrifuged at 12,000× *g* for 30 min at 4 °C. The supernatant was discarded, and the pellet was resuspended using 100 µL of SM buffer (50 mM Tris-HCl [pH7.5], 100 mM NaCl, 8 mM MgSO_4_, 0.01% gelatin; G-Biosciences, St. Louis, MO, USA), and stored at 4 °C until further use.

#### 2.4.4. Preparing Soft Agar

Hard agar plates were prepared using broth media supplemented with 1.5% (*w*/*v*) agar and were used for bacterial culturing and as base layers for soft agar overlay assays. Soft agar overlays were prepared using BHI broth supplemented with 0.5% (*w*/*v*) agar. Following autoclaving and cooling to 55 °C, CaCl_2_ and MgSO_4_ were added to both media to a final concentration of 2.5 mM prior to use.

#### 2.4.5. Spot Assay on Double-Layer Agar

Double-layer agar spot assays were performed by mixing 100 µL of overnight bacterial culture with 3 mL of BHI soft agar and overlaying the mixture onto BHI agar plates. After solidification, 10 µL of concentrated filtrates were spotted onto the bacterial lawn, air-dried, and incubated at 37 °C with or without 5% CO_2_ overnight. Zones of bacterial lysis were collected, resuspended in sterile DPBS, vortexed, centrifuged (12,000× *g* for 5 min), and filtered through 0.22 µm syringe filters. Filtrates were subsequently serially diluted (10-fold) in sterile DPBS and re-screened using spot dilution assays on fresh double-layer agar plates inoculated with the corresponding bacterial host.

#### 2.4.6. Phage Stock Preparation

The double-layer agar assay was done by adding 100 µL of spot filtrate or phage stock to a 1.5 mL tube containing 100 µL of bacterial host overnight culture. This mixture was left to incubate at room temperature for 15–20 min for phage adsorption, then transferred into 3 mL of BHI soft agar, mixed, and poured on top of a regular BHI agar plate, swirling the plate to cover the entire surface with the soft agar top layer. Plates were allowed to solidify at room temperature before being inverted and incubated at 37 °C with 5% CO_2_ overnight. After incubation, plates with between 30 and 300 plaques were selected for calculating phage stock titters in PFU/mL. Isolated plaques were transferred into 500 µL of SM buffer, filtered using a 0.22 µm syringe filter and stored at 4 °C.

#### 2.4.7. Phage Enrichment and Serial Enrichments

Sample enrichments and serial enrichments were accomplished by inoculating broth with target bacterial host and adding 1 mL or 1 g of samples to the mixture. For a single enrichment, 50–200 µL of overnight bacterial culture in BHI broth were added to 5 mL BHI broth in 15 mL centrifuge tubes and incubated at 37 °C shaking 100 rpm overnight. A total of 50 µL of each was added when more than one isolate was used to inoculate into the broth. After a single isolate was used 100 µL of culture was added. For those bacteria with longer latent growth phases, 200 µL of culture were added to the broth. After incubation, tubes were centrifuged at 3900× *g* for 15 min at 4 °C then supernatants were filtered using a 0.22 µm syringe filter and stored at 4 °C. For serial enrichment, 1 mL of the unfiltered supernatant from the first enrichment step was added to a new 15 mL centrifuge tube containing 5 mL of BHI broth and the bacterial inoculum and incubated at 37 °C shaking at 100 rpm overnight. This was repeated four times, after the final incubation; then the tubes were centrifuged at 3900× *g* for 15 min at 4 °C and supernatants were filtered using a 0.22 µm syringe filter.

### 2.5. Bacterial Inoculation

To prepare bacterial inoculums, a new culture was started from cryopreserved glycerol stocks in 5 mL of BHI broth and incubated at 37 °C with a shaking speed of 220 rpm overnight. One hundred µL of the overnight culture was used to inoculate enrichment tubes and 3 mL soft agar-containing tubes.

### 2.6. Bacteriophage Screening

The initial phage-screening assays used were meant to target specific bovine pathogens described previously as bacterial hosts (Table 1), but no phage was detected against any of the pathogenic strains screened through any of the screening methods used. Therefore, we decided to start culturing commensal bacteria from the samples themselves, using those isolates as the host targets for phage screening by using the same samples where they were isolated from. For example, processed RF filtrates were used as the source samples to screen for phages against the bacteria isolated from the same RF pools. The techniques and protocols used to screen phages using animal, water, or soil samples were adapted to improve on challenges and unsuccessful results obtained, culminating in a final protocol that was successful in discovering phages from animal samples when commensal bacteria strains were used as the host targets for the phage screening. All the methods and the gradual improvement procedures are described below. Of note, to improve clarity and facilitate the comprehension of the procedures and flow of the description of each step, we decided to use “Method”. The different methods listed below may reflect the changes involved in the addition of different bacterial hosts, or the addition or removal of a step involved in enrichment or centrifugation, or the addition of different sample types to be screened. The different methods described below do not always refer to the significant changes made in the fundamental principles or in the major steps of a phage-screening method.

#### 2.6.1. Optimization of Bacteriophage-Screening Methods

*A Summary of the Initial Different Methods Applied (Methods 1–11)*

A series of iterative approaches (Methods 1–11) were evaluated to optimize bacteriophage screening from cattle-associated and environmental samples. Initial screening efforts (Methods 1–8) primarily targeted bovine pathogens, particularly *Trueperella pyogenes*, using ruminal fluid (RF), wastewater, liver biopsy, DNS, and other cattle-associated samples. Early methods involved centrifugation, filtration, double-layer agar spot assays, and soft agar overlays supplemented with CaCl_2_ and MgSO_4_. Additional modifications included the use of concentrating pipette systems, enrichment cultures, PEG precipitation, and ultracentrifugation to improve viral particle recovery and phage detection sensitivity.

Despite these methodological adjustments, no detectable phage activity was observed against the pathogenic bacterial hosts screened, including *T. pyogenes*, *Mannheimia haemolytica*, and *Pasteurella multocida*. Ruminal fluid processing presented substantial technical challenges because of its high viscosity and digesta content, frequently causing filter clogging and reduced filtration efficiency.

A substantial methodological shift was introduced in Method 9, in which commensal bacterial isolates obtained from the same ruminal fluid sample pools were used as candidate phage hosts instead of exclusively targeting known pathogens. Using this approach, the first successful phage isolation was achieved from RF samples collected from heifers supplemented with *Megasphaera elsdenii*. The bacterial host was identified as *Escherichia coli*, and the isolated phage belonged to the Caudoviricetes class.

Building on this result, Method 10 incorporated an enrichment step prior to plating using the same commensal host isolates and RF filtrates, resulting in the isolation of eight phages against commensal bacterial strains. In Method 11, enrichment cultures were further modified by incorporating cocktails of bacterial isolates (3–6 isolates per bacterial species) to increase the range of potential host interactions while reducing sample processing requirements. This final approach expanded sample sources to include RF, DNS, VS, US, OS, and liver abscess material. Although this strategy improved workflow efficiency and broadened host exposure, no phages were successfully isolated against the pathogenic bacterial hosts tested, including *M. haemolytica*, *P. multocida*, *T. pyogenes*, *Mycoplasma bovis*, and *Moraxella bovoculi*.

*Method 12*

For this method, serial enrichments were used instead of a single enrichment step, as used in previous methods. This change was made to enhance the phage detection rate as the initial sample volume was relatively small and thus there might be low phage cell numbers in the sample screened. The same samples used in the previous method (Method 11) (RF, DNS, VS, US, OS, and liver abscess) were screened again in this method plus the addition of soil samples.

In addition to the pathogen hosts (*M. haemolytica*, *P. multocida*, *T. pyogenes*, *M. bovis*, and *M. bovoculi*), commensal isolates obtained from the different samples were also used as target hosts for the serial enrichments. For the known bacterial hosts (pathogen hosts), their growth phase was determined by measuring the optical density (OD_600_) over time during incubation at 37 °C. Then, pathogen inoculums were used when their cell growth was at the logarithmic phase for the serial enrichments and double-layer agar assays. The original samples were combined in pools as described previously prior to culturing, and these pools were added to the first enrichment tube without any filtering being done prior to it, and 250 µL of chloroform was added to the 5 mL BHI enrichment broth (only for the first enrichment).

**Figure 1:**
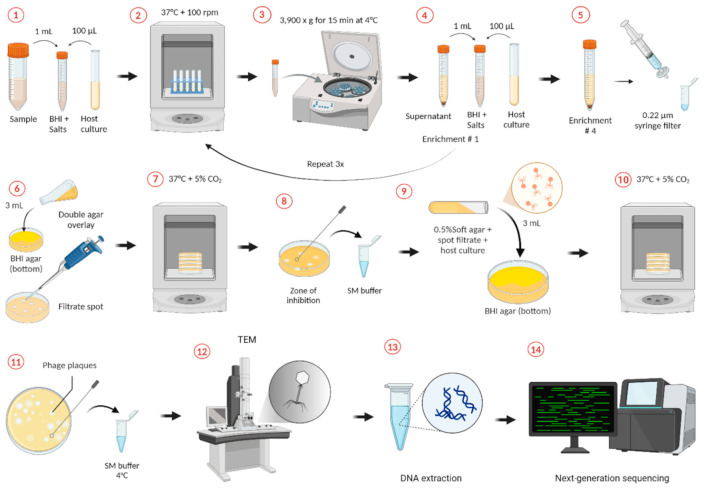
Optimized phage-screening method (Method 12) using serial enrichments from animal origin samples. ① Initial enrichment using initial sample and target host bacteria in BHI broth with the addition of CaCl_2_ and MgSO_4_. ② Incubation step in a shaking incubator set to 37 °C and 100 rpm. ③ Centrifugation step following the incubation step where centrifuge tubes are spun at 3900× *g* for 15 min at 4 °C. ④ Centrifuge tubes removed from the centrifuge are used for following enrichment where supernatants and target host bacteria are combined in BHI broth with the addition of CaCl_2_ and MgSO_4_. Once centrifugation is done, the first enrichment is complete, and steps 2–4 are repeated 3 more times for a total of 4 enrichments. ⑤ After the fourth enrichment is complete, supernatants are filtered with a 0.22 µm syringe filter into 2 mL tubes kept at 4 °C. ⑥ Double-layer agar spot assay. ⑦ Incubation of spot assay plates at 37 °C + 5% CO_2_ (CO_2_ optional). ⑧ Zone of inhibition due to lytic activity of phages in the filtrates picked with a loop and dispensed into SM buffer for a presumptive phage stock. ⑨ Double-layer agar assay to obtain isolated plaques from a mixture of 0.5% soft agar + bacterial host culture (100 µL) + presumptive phage stock from the spot assay (100 µL). ⑩ Incubation of double agar assay plates at 37 °C + 5% CO_2_ (CO_2_ optional). ⑪ Selection and transfer of isolated plaques into SM buffer for long-term storage at 4 °C. ⑫ Transmission electron microscopy (TEM) of phage particles. ⑬ Genomic DNA extraction from isolated phages. ⑭ Whole genome sequencing and analysis of phages. Created in BioRender.

The serial enrichment method proved to be the optimal method among all methods that we tested for the discovery and isolation of phages from animal and soil samples against commensal bacterial isolates. Chloroform was initially added to reduce the bacterial load of samples in the absence of a filtration step. However, because chloroform may reduce the viability of lipid-containing or enveloped phages and potentially bias recovery toward non-enveloped phages, this step was subsequently removed from the protocol. All other steps were kept as before. Using this updated procedure in Method 12 (Figure 1), bovine fetal samples were tested for the first time. The fetal samples included fetal ruminal fluid and meconium. Commensal bacteria were cultured from these samples, and the serial enrichment protocol was used to screen for phages.

#### 2.6.2. High-Throughput Phage-Screening Assay

In addition to the 12 different methods described above, a high-throughput screening protocol enabling the phage screening of multiple samples at the same time was also tested. This method was adapted from Olsen et al. [57]. For this, different samples including DNS, OS, VS, TR, and RF were screened. Upon arrival at the lab, NS and TR swabs were pooled and transferred to a tube for each sample type containing 20 mL of DPBS, while VS and OS samples were pooled, each in a tube containing 10 mL of DPBS. All tubes were vortexed thoroughly, centrifuged at 3900× *g* for 15 min at 4 °C; then supernatants were transferred into 2 mL centrifuge tubes and centrifuged at 20,000× *g* for 10 min at 4 °C. The supernatants were filtered using 0.22 µm syringe filters, concentrated using PEG6000 + NaCl solution, and stored in 2 mL cryovials containing 500 µL glycerol + 1000 µL sample filtrate concentrate.

The selected bacterial hosts used as the targets of the high throughput phage screening were *T. pyogenes*, *M. haemolytica*, *P. multocida*, and *M. bovis*. Additionally, a positive control was used to determine the efficacy of the protocol by spiking a sample with a known *E. coli* phage and testing that sample against an *E. coli* isolate. Culture negative samples were also used as negative controls.

Samples were firstly added to each well (a different sample per well), 2 mL 96-well plates; the initial volume of samples (frozen concentrate filtrates) used varied from 500 to 1000 µL. Then, 37 µL of CaCl_2_ (0.5 mM), 37 µL of MgSO_4_ (0.5 mM), 636 µL of BHI broth 4.4×, and 90 µL of bacterial host culture were added to each well, except for the positive and negative controls. After adding all components, BHI 4.4× was added to complete the volume of wells with 500 and 750 µL of samples, matching the level of the ones with a 1000 µL sample volume; then the contents of each well were mixed by pipetting up and down before the plate was sealed with a foil film. Plates were incubated at 37 °C + 5% CO_2_ overnight. After incubation, the contents of each well were transferred into 2 mL microcentrifuge tubes using a multichannel pipette, then centrifuged at 15,000× *g* for 2 min at 4 °C. The supernatants were filtered with a 0.22 µm syringe filters into new 2 mL deep well 96-well plate, sealed with film, and stored at 4 °C until used.

A total of 200 µL of overnight host bacteria broth culture was mixed into 6 mL of soft BHI agar and poured on top of a rectangular Petri dish (127.76 mm × 85.48 mm) containing TSAb hard agar and another containing BHI hard agar, covering the entire surface, and left to solidify before the next step. Filtrates were then spotted using a 96-needle applicator on both BHI and TSAb plates by immersing the applicator in the 96-well plate and gently pressing it on the soft agar overlay. The applicator was sterilized with 100% ethanol and flamed between each use and plates were incubated at 37 °C overnight.

### 2.7. Phage and Bacterial Host Genome Sequencing

#### 2.7.1. Bacterial Host DNA Extraction

The genomic DNA from selected bacterial host isolates was extracted as previously described [58] for whole genome sequencing using a DNeasy blood and tissue kit (Qiagen, Inc., Hilden, Germany) from broth cultures. Additionally, genomic DNA was extracted from bacterial phage hosts using a Quick-DNA Fungal/Bacterial Miniprep Kit (Zymo Research, Irvine, CA, USA) following the manufacturer’s instructions, with minor modifications [59], for taxonomic identification.

#### 2.7.2. Phage DNA Extraction

Phage DNA was extracted using the Norgen Biotek phage DNA extraction kit (Norgen Biotek, Thorold, ON, Canada) following the manufacturer’s instructions with a few modifications as previously described [60]. A pre-treatment step was performed to remove bacterial genetic material from the samples prior to extracting phage DNA. Briefly, 450 µL of filtered supernatant-containing phage was treated with 50 µL of DNase I (1 U/µL; Qiagen) and 1 µL of RNase A (10 mg/mL; Qiagen) and incubated for 90 min at 37 °C. Next, 20 µL of Ethylenediaminetetraacetic acid (EDTA) at a concentration of 20 mM was added with the goal of inactivating the activity of the DNase and RNase enzymes, and lastly, 1.25 uL of Proteinase K (20 mg/mL) was added and the mix was incubated at 56 °C for 90 min. For the extraction of genomic DNA from the 1RFP6A and 3RFP5C_6 phages however, a bead-beating lysis method was employed prior to genomic DNA extraction with the Zymo Quick-DNA Miniprep kit (Zymo Research Corporation, Irvine, CA, USA).

#### 2.7.3. DNA Concentration and Quality Assessment

Next, a Nanodrop spectrophotometer (Thermo Fisher Scientific, Waltham, MA, USA) was used to assess the quality of the extracted genomic DNA samples, and a Qubit 4 fluorometer (Thermo Fisher Scientific) with the double-stranded DNA (dsDNA) high-sensitivity assay kit (Thermo Fisher Scientific) was used to quantify the extracted genomic DNA.

#### 2.7.4. Sanger Sequencing and Taxonomic Identification of Bacterial Hosts

Sanger sequencing of the 16S rRNA gene was used for the taxonomic identification of bacterial host isolates. A PCR reaction containing 10 µL of iQ Supermix (Bio-Rad Laboratories Inc., Hercules, CA, USA), 1 µL of 27F primer (5′-AGAGTTTGATCMTGGCTCAG-3′; 10 µM), 1 µL of 1492R primer (5′-TACGGYTACCTTGTTACGACTT-3′; 10 µM), 2 µL of DNA template, and 6 µL of nuclease free water (Corning, Corning, NY, USA) to complete a reaction volume of 20 µL was used to amplify the near-full-length 16S rRNA gene. The cycling conditions were as follows: an initial denaturation step of 95 °C for 5 min; followed by 35 cycles of 95 °C for 45 s, 50 °C for 30 s, and 72 °C for 2 min; and a final elongation step at 72 °C for 5 min. This was done in an Eppendorf Mastercycler (Eppendorf, Hamburg, Germany). After amplification, PCR products were sequenced by the Molecular Cloning Laboratories (MCLAB, San Francisco, CA, USA), and sequences were identified with the Basic Local Alignment Search Tool (BLAST https://blast.ncbi.nlm.nih.gov/Blast.cgi; accessed on 5 March 2025); and the non-redundant National Center for Biotechnology Information (NCBI) nucleotide database.

#### 2.7.5. Whole-Genome Sequencing and Annotation

*Phages*

Libraries were preprepared for all phage genomic DNA using the Qiagen FX DNA library preparation kit (Qiagen, Germantown, MD, USA), except for phages 1RFP6A and 3RFP5C_6 which had DNA libraries prepared with the Illumina DNA Prep kit incorporating UDI 10 bp indices (Illumina, Inc, San Diego, CA, USA). An Illumina MiSeq instrument was used for phage genome sequencing utilizing the MiSeq v2 reagent kit (500 cycles; Illumina), as for the phages 1RFP6A and 3RFP5C_6, libraries were loaded into an Illumina NovaSeq 6000 instrument equipped with an S4 flow cell (300 cycles).

For all phages, sequences quality control and adapter trimming were accomplished with the Trim Galore v. 0.6.10 [61] (https://github.com/FelixKrueger/TrimGalore, accessed on 5 March 2025) tool, and genomes were assembled using Shovill v. 1.1.0 (https://github.com/tseemann/shovill, accessed on 5 March 2025) pipeline. Then, the quality of the assemblies was evaluated using QUAST [62] and annotation conducted through Pharokka v. 1.1.0 [63]. Additionally, genomes were screened for the presence of virulence factors using the bacterial virulence factor database (VFDB) [64], antimicrobial resistance genes through the comprehensive antibiotic resistance database (CARD) [65], and for the presence of tRNA genes with the assistance of the tRNAscan-SE online resource (http://trna.ucsc.edu/tRNAscan-SE/, accessed on 5 March 2025). Additionally, the formula used to determine the sequencing coverage was coverage = (number of reads × read length)/genome length.

Each phage had its genome compared to those available in the NCBI database using the BLASTn (https://blast.ncbi.nlm.nih.gov/Blast.cgi, accessed on 5 March 2025). tool for taxonomical identification. The combination of percent identity and percent coverage were used to define the species, genus, family, or class of the phages based on their sequence similarity (% identity × % coverage). Higher sequence similarities resulted in better classification for the phages, considering sequence identities of higher or equal to 95% to a species level, between 70 and 95% to the genus level, or less than 70% to the family level [66].

*Bacterial hosts*

Genomic DNA libraries from bacterial hosts were prepared using an FX DNA library preparation kit (Qiagen) and sequenced in an Illumina MiSeq instrument (Illumina) with the MiSeq v.2 reagent kit (500 cycles; Illumina) following the manufacturer’s instructions. The fastp v.0.23.2 [67] tool was used to do quality filtering and adapter trimming of sequence reads; additionally, reads that had a mean Phred quality score smaller than 15 with a sliding window of 4 bp and/or were shorter than 100 bp were removed from further analysis. Next, genomes were de novo assembled with SPAdes v.3.15.5 [68] with the isolate option activated. The BBMap v.38.96 aligner (https://sourceforge.net/projects/bbmap, accessed on 5 March 2025) [69] was used to remove contigs that were shorter than 500 bp using the minlength parameter configured to 500 bp. Next, contigs completeness and possible contamination were assessed using the lineage-specific workflow of CheckM v.1.2.0 [70]. Finally, QUAST v.5.2.0 [62] was used to assess assembly qualities and the Prokaryotic Genome Annotation Pipeline (PGAP) v.2022-10-03.build 6384 [71] from NCBI was used to annotate the genomes. The GTDB-Tk v.2.1.1 classify workflow with the Genome Taxonomy Database (GTDB) release 207 was used to define each bacterial genome taxonomically [72,73]. All parameters for software or bioinformatics tools were set to default unless otherwise specified.

### 2.8. Transmission Electron Microscopy Imaging of Phage Cells

Transmission electron microscopy (TEM) was performed on a subset of 11 representative phage isolates selected based on the timing of isolation, host diversity, and availability of purified high-titer lysates. Due to the substantial time and cost associated with TEM imaging, it was not feasible to image all 83 isolated phages. Phage cell suspensions were prepared by propagating phages on BHI double-layer agar plates containing a lawn of bacterial cell host. A 10 µL loop was used to streak spread a phage stock solution onto the soft agar overlay prior to incubation at 37 °C + 5% CO_2_ overnight. After incubations, the areas where bacterial lysis were observed on the overlay soft agar were removed and added to 500–1000 µL of nuclease free water (Corning, Corning, NY, USA), centrifuged at 12,000× *g* for 20 min at 4 °C, the supernatants were then filtered with 0.22 µm syringe filters, and filtrates submitted for imaging at the NDSU Electron Microscopy Center (Fargo, ND, USA). A drop of sample was placed on a 300-mesh formvar-carbon-coated copper grid (Ted Pella Inc., Redding, CA, USA) for two minutes and wicked off with filter paper. The negative stain phosphotungstic acid 0.1% was added to the samples, the pH was adjusted to 7–8, then stained samples were dropped onto the grid and allowed to stand for two minutes and wicked off and the grid was allowed to air-dry. High-resolution analytical TEM was accomplished using a JEOL JEM-1400 Flash transmission electron microscope (JEOL USA, Peabody, MA, USA) at a direct magnification of 50,000× *g*, HV = 120 kV, and a NANOSPTRT15 camera with 800 (ms) exposure × 2 standard frames, gamma of 1, no sharpening, and normal contrast.

## 3. Results

### 3.1. Overview of the Samples Screened for Phage and Their Origin

A total of 1214 samples collected from seven different beef cattle trials (98.4%), and the environment were screened for the presence of phages against bovine pathogenic bacteria and commensal bacteria originated from cattle gut, respiratory and reproductive tracts, and bovine fetuses in the present study (Table 2). Those cattle-origin samples were consisted of ruminal fluid (*n* = 799), feces (*n* = 30), and deep nasopharyngeal (*n* = 130), vaginal (*n* = 124), uterine (*n* = 15) and ocular swabs (*n* = 34), and liver biopsy tissue from healthy beef cattle (*n* = 10), liver abscesses (*n* = 3) from beef cattle, and raw milk from dairy cattle (pooled; *n* = 2), and soil cores (*n* = 12), runoff water from beef cattle facility (*n* = 2), beef cattle drinking water (*n* = 2), and municipal wastewater (*n* = 4) (Table 3).

### 3.2. Evolution and Assessment of the Phage-Screening Methods

The use of commensal bacterial isolates as target hosts and the inclusion of an enrichment step to the phage-screening process were considered as the first and second successful modifications from the initial tests, respectively. Therefore, a further improvement on the enrichment step was made for Method 12, and this improvement was performing four rounds of serial enrichment. In this method, both bovine bacterial pathogens and commensal bacterial strains were used as phage hosts, and a total of 67 phages were isolated. All of these phages inhibited the commensal bacterial hosts tested. No phage against pathogenic bacterial hosts was detected.

From the two runoff water samples, two drinking water samples, 30 fecal, 28 DNS, 62 RF, 12 soil, and one VS samples that were screened by this method, 16 phages from feces, one from DSN, 45 from RF, and five from VS samples were isolated (Table 3).

Serial enrichments of sample filtrates were found to be the most effective procedures that enhanced the phage detection rate from both cattle and environmental samples. However, the addition of chloroform in the first enrichment round of the four serial enrichments used was later recognized as unnecessary and that it may compromise the phage detection outcomes. The original purpose of adding chloroform in this early step of enrichment was to lysate bacterial cells to release possible phage particles from the infected host cells (intracellular), and to reduce the unrelated bacterial cell load present in the screened samples. However, chloroform could also degrade and reduce the stability of some phages, in addition to increasing the quantity of cell debris from lysed bacterial cells. Therefore, we removed this step involved in chloroform addition during the enrichment step. By this modified method (Method 12), two more phages from the RF (*n* = 20 screened), and one phage from the fetal RF (*n* = 20) were recovered. The 19 fetal meconium samples screened did not yield any phage detection. No phages were detected against any bovine pathogens tested in this method.

### 3.3. High-Throughput Screening Method

For the high-throughput 96-well plate method, there was a total of 109 samples used, including 22 RF, NS, TR, and VS, and 21 OS samples, collected in seven different days. Sample filtrates were used in soft agar lawns inoculated with the *M. haemolytica*, *P. multocida*, *M. bovis*. No lysis was observed against any of the tested pathogens.

Overall, among the different phage-screening methods that we used, Method 12 was found to be the optimal assay that enabled us to identify phages from different sample types obtained from different cattle body sites against ruminal and other commensal bacteria. After removing the chloroform addition step during enrichment, the updated Method 12 was chosen as the most effective method for screening different microbial samples obtained from different cattle body sites including ruminal fluid, fecal, vaginal and nasopharyngeal, and fetal intestine for phage isolation against commensal bacteria. This method involves four enrichment steps.

### 3.4. Isolated Phages and Their Respective Bacterial Hosts

From the 1214 samples screened for phages, through all of the different tested methods (13 different methods) described in this study, a total of 83 phages were isolated and banked, the equivalent of 6.8% of total samples screened. Moreover, 59 RF samples (7.4% phage recovery rate from samples screened), 15 of 30 fecal samples (50%), seven VS (5.6%), one DNS (0.8%), and one fetal RF (5%) resulted in phage recovery (Table 3). The percentage numbers are only an overall view and any inference done over it should be interpreted cautiously, because there were different methods used, different bacterial hosts (pathogens vs. commensals), and the number of samples pooled together for phage screening. However, fecal samples followed by RF samples showed the highest phage recovery rate (Table 3). Overall, most of the phages isolated in the present study were lytic against bacteria species within the *Bacillus* genus, making up 41% of total phages isolated (Table 4). The second most common bacterial host was *Escherichia/Shigella* (9.6%), followed by *Shouchella* spp. (6%), *Corynebacterium* spp., and *Lysinibacllus* spp. (4.8%), and *Caldibacillus* spp. (3.6%) (Table 4). However, we were not able to identify any phages against bovine pathogens such as *T. pyogenes*, *M. haemolytica*, *P. multocida*, and *M. bovis*.

### 3.5. Taxonomic Characterization of Bacterial Hosts and Whole-Genome Sequencing of Phages and Their Respective Bacterial Hosts

Due to budgetary and sequencing cost limitations, only a subset of representative phages (*n* = 11) (Table 5), selected based on host range, sample origin, and plaque morphology diversity, was subjected to whole-genome sequencing. These 11 phages were isolated from bovine rumen (*n* = 10) and vagina (*n* = 1). They were lytic phages, and their bacterial hosts were *Alkalihalobacillus clausii* (*n* = 1), *Bacillus safensis* (*n* = 4), and *E. coli* (*n* = 6). The genome results for these 11 phages have been reported as Genome Announcement as shown in Table 5. [74]. Briefly, the genome sizes of these bacteriophages showed considerable variation, ranging from 20,216 base pairs (bp) to 168,609 base pairs (bp) and the GC content (%) varied from 33.94 to 44.35. Analyzing the genomes, it was found that none contained CRISPR (Clustered Regularly Interspaced Short Palindromic Repeats) sequences, tmRNAs (transfer-messenger RNAs), virulence factors, or antimicrobial resistance genes [74]. The absence of *tmRNAs*, antimicrobial resistance genes, and virulence factors ensure there are no risks of transferring these pathogenic traits to host bacteria or other members of the microbiome once phages are replicated. All the phages sequenced were tailed phages and belonged to seven different taxonomy classifications: *Caudoviricetes* (*n* = 3), *Felixounavirus* spp. (*n* = 2), *Tequatrovirus* spp. (*n* = 2), *Vequintavirus* spp. (*n* = 1), *Salasmaviridae* family phage (*n* = 1), *Herelleviridae* family phage (*n* = 1), and *Siophivirus* spp. (*n* = 1).

### 3.6. Morphological Characterization

**Figure 2:**
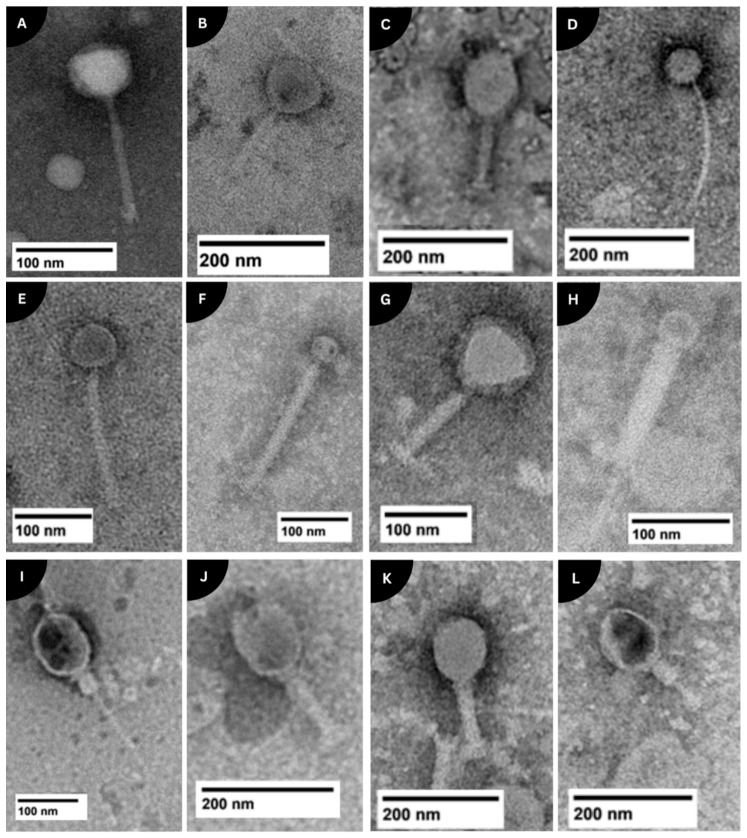
Transmission electron microscopy images of phages (host) (**A**) 1RFP6A (*E. coli*), (**B**) 2RFP1H_AA (*E. coli)*, (**C**) 2RFP1A2_1 (*E. coli)*, (**D**) 2RFP1E_AA (*A. clausii*), (**E**) 2RFP5B2_3 (*B. safensis*), (**F**) 2RFP8A2_3 (*B. safensis)*, (**G**) 2RFP1C2_AA (*E. coli*), (**H**) 3RFP5C_2 (*B. safensis)*, (**I**,**J**) TP1813_CB_A_EVS_2 (*E. coli*), and (**K**,**L**) TP167_CB_C_ERF_3 (*E. coli)* isolated from animal origin samples with scales adjusted to nm.

Phages were characterized by TEM and phage cells displayed diverse morphologies, but all imaged phages were within the *Caudoviricetes* class of tailed phages and had icosahedral heads (Figure 2). The phages displayed contractile tails as shown in Figure 2I,J, where the phages TP1813_CBA_EVS_2 and TP167_CB_C_ERF_3 can be seen with their elongated and contracted tail. Through TEM, it is also possible to observe the similar size and structures of phage TP1813_CBA_EVS_2, isolated from the vaginal swab, and phage TP167_CB_C_ERF_3, obtained from the ruminal fluid of heifers. The phage particle sizes ranged from ~180 to 260 nm, with head sizes ranging from ~40 to 100 nm and tail lengths were ~100 to 210 nm. The high contrast in TEM allowed for the visualization of fine structural details, such as tail fibers and sheath striations, providing insight into the diversity and morphology of these bacteriophages.

## 4. Discussion

In the present study, we screened a total of 1194 samples obtained from different cattle body sites, including the gut, respiratory and reproductive tracts, and fetal gut, as well as environmental sources (*n* = 20), using 13 phage detection methods. Through incremental modifications, such as the addition or removal of steps in the protocol, incorporating bacterial host samples, or expanding the variety of sample types to be screened, we optimized a phage-screening method that allowed us to isolate 83 phages against commensal bacteria. Overall, with Method 12, the highest number of phages (81% of the total phages isolated) was recovered, and thus displayed the potential to be the most suitable method for screening phages from bovine gut, respiratory, reproductive, and fetal-associated microbial samples.

Among the different bovine and environmental samples screened, a greater number of phages were recovered from the ruminal fluid samples screened (*n* = 75). The rumen is a complex ecosystem harboring bacteria, methanogenic archaea, fungi, viruses and protozoa [41]. Viral cells including bacteriophages are relatively high in quantity in the rumens [41]. The ruminal phages can interact with bacteria cells in the rumen either through lysogenic or lytic cycles, and their population in the rumen can be influenced by ruminal bacterial compositions [75] and fermentation characteristics (VFA, pH), as well as diet [76]. Despite the rumen being reported to contain a relatively high bacteriophage cell population [41], the phage isolation rate per sample screened was lower than that of fecal samples. This might be due to several factors. One of which is associated with the ruminal bacterial hosts used in the present study. These bacterial hosts were isolated under aerobic culturing conditions given the challenge associated with conducting phage-screening procedures under a strict anaerobic condition. The anaerobic nature of the rumen may be limited in identifying and isolating even more phages from the ruminal fluid samples screened.

In the present study, we isolated phages that are lytic to commensal bacterial species within *Bacillus*, *Escherichia/Shigella*, *Shouchella*, *Corynebacterium*, and *Lysinibacillus* genera originated from the cattle rumen. *Bacillus* species, such as *Bacillus licheniformis* and *Bacillus subtilis*, have been explored as probiotic supplements in cattle [77,78]. As these *Bacillus* spp. can form spores and be able to survive in the anaerobic environment of the rumen [79,80]. Additionally, some *Bacillus* spp. have been shown to inhibit the growth of certain pathogens [81,82,83], and thereby improve gut health. Furthermore, *Bacillus* species have been reported to improve feed efficiency by enhancing starch and fiber digestibility [84,85,86,87,88]. We isolated phages against ruminal *Bacillus* spp. in the present study, and the impact of modulation of the ruminal *Bacillus* population using the bacteriophages we isolated on ruminal microbiome composition, fermentation and feed digestion deserves further research.

*Shouchella* spp. and *Escherichia/Shigella* spp. primarily contribute to protein metabolism, can hydrolyze cellulose and produce H_2_, but have not been directly linked to methanogenesis [89,90]; although, their interactions within the ruminal microbiome may influence overall fermentation dynamics. Some of the *Escherichia/Shigella*, and *Citrobacter/Klebsiella/Salmonella* species have been reported to be negatively correlated with ruminal fermentation [91,92]. Thus, the phages isolated against *Escherichia/Shigella* may be of interest, as these bacterial genera include pathogenic strains and have previously been associated with altered ruminal fermentation efficiency. Although the effects of these phages on ruminal microbial ecology and feed digestion were not evaluated in the present study, they may warrant future investigation for their potential role in modulating ruminal bacterial populations. Members of the *Shouchella* genus, namely *Shouchella clausii*, formerly known as *Bacillus clausii* [93], have been used as probiotics in humans [94], similarly to *Enterococcus* spp. and *Streptococcus* spp. [95]. Although the *Corynebacterium* genus does encompass pathogenic species such as mastitis-associated *C. amycolatum* and *C. pseudotuberculosis* [96], certain *Corynebacterium* species are associated with amino acid fermentation and nutrient degradation in the rumen [97]. *Lysinibacillus* is one of the least characterized genera in the ruminal microbiota; *Lysinibacillus fusiformis* has been linked with increased feed digestibility and ruminal VFA production and reduced methanogens by limiting H_2_ availability to methanogenic archaea [98]. Given that these bacterial species are positively associated with increased nutrient utilization and reduced methanogenesis, the phages that we isolated against these commensal bacterial species should be investigated thoroughly. Future studies should focus on isolating phages targeting ruminal bacteria and methanogenic archaea associated with ruminal microbial ecosystems. The optimized Method 12 developed in this study may provide a useful framework for future isolation and screening of phages against diverse ruminal microbial species. We were able to isolate phages from vaginal and nasopharyngeal swab samples; although, the isolation rate was lower compared to fecal and ruminal fluid samples. This is not commonly reported in the literature, except for a few findings where respiratory phages have been isolated from the human throat [99] and lysogenic phages targeting vaginal bacteria were induced. Additionally, reports have explored the use of phage therapy against reproductive infections in cattle [100,101]; however, direct isolation of lytic phages from the nasopharyngeal, vaginal, or uterine microbiomes of cattle remains largely unexplored. Similarly, the presence and role of bacteriophages in the bovine respiratory microbiome remain under-characterized. Given the important role of the nasopharyngeal microbiome in maintaining respiratory health and airway immune response, particularly in high-risk cattle for BRD, microbiome modulation using bacteriophage presents a potential strategy for reducing BDR disease susceptibility [102,103].

Likewise, the female reproductive tract microbiome significantly influences cattle health and fertility [25,104,105]. Specific bacterial groups within the vaginal and uterine microbiomes have been associated with reproductive success or dysfunction, suggesting that microbiome-targeted interventions could enhance reproductive efficiency [26,104].

A limitation encountered in this study that could have compromised the phage discovery from DNS and VS samples was that samples were collected via swab. The swab-based sample collection has its limitations as the sample volume would be small compared to ruminal fluid or feces. This would result in a reduced chance of phage detection as these swab samples may have a very limited number of phage particles.

Despite this limitation, we were still able to recover phages from the nasopharynx and vaginal canal of cattle. Future studies should modify the sample collection method to ensure a significant biomass is obtained from the vagina and nasopharynx. One alternative method for nasopharyngeal sampling could be a suction pump involved sampling. A bronchoalveolar lavage sampling is also worth testing for phage screening against BRD pathogens. As for the reproductive tract, a vaginal brush-based sampling could enhance phage detection rate. We believe that the four round of serial enrichment steps we applied in Method 12 increased the chances of phage discovery from DNS and VS samples. Thus, increasing the number of enrichment steps even further for the low microbial biomass samples such as DNS and VS should be considered in future studies.

We isolated a phage from the ruminal fluid of a 180-day-old bovine fetus, representing, to the best of our knowledge, the first report of phage isolation from a healthy bovine fetus. This finding provides additional evidence challenging the long-held sterile womb theory [106], which proposed that the gravid uterus and fetus remain free of microbial colonization during gestation. Recent studies have instead suggested that bacterial and archaeal DNA fingerprints in calf intestine may begin prenatally [19,53,107]. The isolation of a bacteriophage from fetal ruminal fluid further raises the possibility that viral colonization may also occur before birth in cattle. Although contamination cannot be fully excluded in low-biomass fetal microbiome studies, several factors support the biological relevance of this observation. Fetal samples were collected under aseptic conditions to the extent feasible within a large-animal setting, and the isolated phage demonstrated active infectivity against its bacterial host rather than representing only residual viral nucleic acid contamination. In addition, emerging evidence from both human and animal studies has reported the presence of microbial DNA and viable microorganisms in fetal-associated tissues and fluids, supporting the plausibility of prenatal microbial and viral exposure [108,109]. Nevertheless, because this study was not specifically designed to conclusively distinguish true in utero colonization from potential contamination, these findings should be interpreted cautiously and validated in future studies using larger sample sizes, rigorous contamination controls, and culture-independent virome approaches. Despite screening a relatively large number of samples (*n* = 1214) and successfully isolating phages against commensal bacteria using Method 12, we were unable to identify phages targeting key bovine phages (*T. pyogenes*, *M. haemolytica*, *P. multocida*, and *M. bovis*). Several ecological and technical factors could have contributed to the unsuccessful phage isolation against pathogenic bacterial hosts. One of the reasons could be associated with the origin of the bacterial hosts used in this study. These pathogenic strains originated from different animals involved in animal trials or from samples submitted to the veterinary diagnostic lab. These pathogenic strains have undergone multiple freeze–thaw cycles and had been cultured in the lab several times prior to being used in the phage screening. All of which might have an influence on their phage susceptibility. Thus, to increase the success rate of isolating phages against these bovine pathogen species, fresh isolation of the pathogenic isolates from the same herd of cattle, or ideally the isolation of pathogenic strains from the same type of samples screened for the phages, should be considered in future studies. The strains used in the present study may not be optimal for phage screening, and their genomes may contain anti-phage defense systems and defense islands [110]. Thus, it is necessary to perform genotyping and whole genome sequencing analysis of these bovine pathogenic species before using them as bacterial hosts for phage screening. In addition, a comprehensive growth curve of the pathogenic bacterial strains should be performed to identify the growth stage that is the most susceptible for phage infection [106].

No phages to date have been identified that can infect the liver abscess and mastitis-associated pathogen *T. pyogenes* or the pinkeye-associated pathogens *M. bovis* and *M. bovoculi*. Prophages, often referred to as temperate phages, have been discovered within the genomes of *M. haemolytica* [107,111] and *P. multocida* [112], which have the potential to be triggered into a lytic cycle, classified as *Siphoviridae* and *Myoviridae*. Temperate phages can significantly contribute to bacterial pathogenesis, virulence, and pathogen evolution through horizontal gene transfer [113]. *M. haemolytica* and *P. multocida* are opportunistic pathogens that are present in the upper respiratory tract of healthy cattle. A limited number of lytic phages have been found for *P. multocida*, including a type-specific one for capsular type D. However, this phage is highly specific, only lysing non-toxigenic type D *P. multocida* and failing to infect any other strains of *P. multocida* [114]. Another phage targeting *P. multocida* exhibited broader activity against capsular types B:2 and A:1 [115,116]. This illustrates the specificity of type-specific *Pasteurella* phages and underscores the challenges associated with sourcing phages for this pathogen from environmental samples. For the future phage screening aimed at BRD bacterial pathogens, a thorough assessment of various types of each *M. haemolytica* and *P. multocida* should be employed to enhance the phage identification. This also indicates that using phage therapy for BRD pathogens may be difficult, necessitating a cocktail of phages for each potential type. In addition to these biological factors, limitations associated with swab-based sampling could also contribute to the unsuccessful detection of phages against screened respiratory pathogens. The opportunistic pathogen *T. pyogenes*, used as a host for the phage screening of VS and an inhabitant of the reproductive tract of cattle, may have been in low abundance in animals sampled in this study because the animals were healthy and without active infections. This further reduced the chances of detecting the pathogen and any associated phages. In summary, the interplay of low pathogen abundance in healthy animals, the limitations inherent in the sampling techniques employed, pooling samples which may have reduced specificity, and the specific health status of the cattle sampled all contribute to the lack of identified phages targeting the pathogens present in the upper respiratory and reproductive tracts.

Screening bacteriophages from environmental or clinical samples presents numerous challenges, particularly when isolating unknown phages. Unlike working with stock phage solutions, which bypass many initial hurdles, successful plaque formation depends on multiple variables. Sampling limitations, along with factors related to screening methodologies, may have an impact on the success of phage screening. A few of these factors include the concentrations of Ca and Mg ions and of soft agar [36], choice of gelling agent in the soft agar overlay [117], the number and concentration of host strains [117,118], and temperature [119,120,121,122]. Furthermore, the addition of certain antibiotics or glycerol to the media can improve plaque formation and visibility by activating the SOS system in bacterial hosts [123]. Some phages require specific cation conditions for adsorption, and the parameters used in this present study may not have been optimal for the selected pathogen hosts. Future studies should test the same samples using optimized methods with varying soft agar and ion concentrations, as divalent cations are crucial for phage DNA penetration into host cells [124,125]. These challenges highlight the need for method development and optimization when isolating phages from animal samples. Novel approaches should refine existing techniques by adjusting these critical factors to improve phage recovery. The lack of phages observed against specific pathogenic bacterial hosts in the present study highlights the need for further refinement of screening parameters, as the tested conditions may not be ideal to support optimal phage–host interactions.

## 5. Conclusions

This study aimed to optimize methodologies for isolating bacteriophages from diverse bovine-associated microbial ecosystems, including the gastrointestinal, reproductive, respiratory, ocular, and fetal environments. Although efforts were made to isolate phages against bovine pathogens such as Trueperella pyogenes, Mannheimia haemolytica, and Pasteurella multocida, the primary outcome of this work was the refinement of bacteriophage-screening and isolation approaches, particularly through the development of the optimized Method 12. Despite the challenges associated with isolating phages against several targeted pathogens, we successfully isolated numerous phages against commensal bacterial hosts originating from the rumen, vagina, uterus, intestinal tract, and other cattle-associated environments. The optimized workflow improved the practicality and throughput of traditional double-layer agar-based phage-screening methods while accommodating the technical challenges associated with processing diverse bovine sample types. Using these approaches, phages were isolated from ruminal fluid, feces, vaginal, nasopharyngeal, ocular, uterine, and fetal ruminal fluid samples, with lytic activity observed against 26 bacterial genera. Overall, this study provides a methodological framework for bacteriophage isolation and preliminary characterization across multiple bovine microbial ecosystems. Future studies should focus on further refinement of isolation strategies, expansion of host range screening, and detailed characterization of phage–host interactions across cattle-associated microbial communities.

## Figures and Tables

**Table 1:** List of bovine pathogen bacterial hosts used in phage screening from animal and environmental samples.

Pathogen Species	Strain ID	Source	Species
*Trueperella pyogenes*	306	Liver abscess	Cattle
*Trueperella pyogenes*	311	Liver abscess	Cattle
*Trueperella pyogenes*	315	Liver abscess	Cattle
*Trueperella pyogenes*	2023_3_60	Ruminal tissue	Cattle
*Trueperella pyogenes*	2022_1_14	Ruminal tissue	Cattle
*Trueperella pyogenes*	2022_1_94	Ruminal tissue	Cattle
*Trueperella pyogenes*	ATCC 19411	Lung	Swine
*Trueperella pyogenes*	22_4153abc	Abscess	Otter
*Trueperella pyogenes*	22_4153lg	Lung	Otter
*Trueperella pyogenes*	22_2564	Perineum	Cattle
*Trueperella pyogenes*	22_2565	Lung	Cattle
*Trueperella pyogenes*	21_2041	Lung	Deer
*Trueperella pyogenes*	7762	Lung	Cattle
*Trueperella pyogenes*	22_2570	Milk	Cattle
*Trueperella pyogenes*	16_8561	Abscess	Cattle
*Trueperella pyogenes*	2645	Abscess	Deer
*Mannheimia haemolytica*	3129	Nasopharynx	Cattle
*Mannheimia haemolytica*	EO_764	Nasopharynx	Cattle
*Mannheimia haemolytica*	EO_700_1	Nasopharynx	Cattle
*Mannheimia haemolytica*	EO_744_2	Nasopharynx	Cattle
*Mannheimia haemolytica*	EO_728_2	Nasopharynx	Cattle
*Mannheimia haemolytica*	EO_755_7_A	Nasopharynx	Cattle
*Mannheimia haemolytica*	EO_748_7_B	Nasopharynx	Cattle
*Mannheimia haemolytica*	EO_749_-1	Nasopharynx	Cattle
*Mannheimia haemolytica*	EO_744_-1	Nasopharynx	Cattle
*Mannheimia haemolytica*	EO_786_-1	Nasopharynx	Cattle
*Mannheimia haemolytica*	EO_777_28_C	Nasopharynx	Cattle
*Mannheimia haemolytica*	EO_766_28_A	Nasopharynx	Cattle
*Mannheimia haemolytica*	EO_777_42_B	Nasopharynx	Cattle
*Mannheimia haemolytica*	EO_716_28_A	Nasopharynx	Cattle
*Mannheimia haemolytica*	EO_760_28_A	Nasopharynx	Cattle
*Mannheimia haemolytica*	EO_712_42_A	Nasopharynx	Cattle
*Mannheimia haemolytica*	EO_764_42_A	Nasopharynx	Cattle
*Mannheimia haemolytica*	EO_704_28_B	Nasopharynx	Cattle
*Mannheimia haemolytica*	EO_751_42_B	Nasopharynx	Cattle
*Mannheimia haemolytica*	EO_735_42_A	Nasopharynx	Cattle
*Mannheimia haemolytica*	EO_786_42_A	Nasopharynx	Cattle
*Pasteurella multocida*	EO_730_42_A	Nasopharynx	Cattle
*Pasteurella multocida*	EO_785_1_B	Nasopharynx	Cattle
*Pasteurella multocida*	EO_720_42_A	Nasopharynx	Cattle
*Pasteurella multocida*	EO_769_28_A	Nasopharynx	Cattle
*Pasteurella multocida*	EO_716_28_A	Nasopharynx	Cattle
*Pasteurella multocida*	EO_763_42_A	Nasopharynx	Cattle
*Pasteurella multocida*	EO_777_-1	Nasopharynx	Cattle
*Pasteurella multocida*	EO_730_1	Nasopharynx	Cattle
*Pasteurella multocida*	EO_739_28_A	Nasopharynx	Cattle
*Pasteurella multocida*	EO_716_2	Nasopharynx	Cattle
*Pasteurella multocida*	EO_718_1	Nasopharynx	Cattle
*Pasteurella multocida*	EO_746_28_B	Nasopharynx	Cattle
*Pasteurella multocida*	EO_777_28_B	Nasopharynx	Cattle
*Pasteurella multocida*	EO_730_42_A	Nasopharynx	Cattle
*Pasteurella multocida*	EO_753_28_A	Nasopharynx	Cattle
*Pasteurella multocida*	EO_787_2	Nasopharynx	Cattle
*Pasteurella multocida*	EO_740_42_A	Nasopharynx	Cattle
*Pasteurella multocida*	EO_712_42_B	Nasopharynx	Cattle
*Pasteurella multocida*	EO_751_42_A	Nasopharynx	Cattle
*Pasteurella multocida*	EO_777_42_A	Nasopharynx	Cattle
*Pasteurella multocida*	2873	Nasopharynx	Cattle
*Escherichia coli*	BHI_cont	Environmental	Environmental
*Moraxella bovis*	7116	Ocular swab	Cattle
*Moraxella bovis*	194735-2	Ocular swab	Cattle
*Moraxella bovis*	3481-2	Ocular swab	Cattle
*Moraxella bovis*	19.13095-6	Ocular swab	Cattle
*Moraxella bovis*	198338-1	Ocular swab	Cattle

**Table 2:** List of bacteriophages isolated from bovine samples through different studies at NDSU and their respective bacterial hosts.

Study No.	Sample ^1^	Host ID	Host Taxonomic ID	Phage ID	Microscopy
2	RF	1RFP6A	*Escherichia coli*	1RFP6A	Yes
2	RF	2RFP1A2	*Escherichia coli* O20:H8	2RFP1A2_1	Yes
2	RF	2RFP1C2	*Escherichia coli* O50:H5	2RFP1C2_AA	Yes
2	RF	2RFP1E	*Alkalihalobacillus clausii*	2RFP1E_AA	Yes
2	RF	2RFP1H	*Escherichia coli* O20:H8	2RFP1H_AA	Yes
2	RF	2RFP5B2	*Bacillus safensis*	2RFP5B2_3	Yes
2	RF	2RFP8A2	*Bacillus safensis*	2RFP8A2_3	Yes
2	RF	3RFP5C	*Bacillus safensis*	3RFP5C_2	Yes
2	RF	3RFP5E	*Bacillus safensis*	3RFP5E_6	Yes
3	RF	AFRI 1_RF_P1A	*Bacillus* spp.	AFRI 1_RF_P1A_1	No
3	RF	AFRI 1_RF_P1C	*Alkalihalobacillus* spp.	AFRI 1_RF_P1C_1	No
3	RF	AFRI 1_RF_P1D	*Bacillus* spp.	AFRI 1_RF_P1D_1	No
3	RF	AFRI 1_RF_P1E	*Microbacterium* spp.	AFRI 1_RF_P1E_1	No
3	RF	AFRI 1_RF_P1G	*Bacillus* spp.	AFRI 1_RF_P1G_1	No
3	RF	AFRI 1_RF_P2A	*Staphylococcus* spp.	AFRI 1_RF_P2A_1	No
3	RF	AFRI 1_RF_P2B	*Cellulosimicrobium* spp.	AFRI 1_RF_P2B_1	No
3	RF	AFRI 1_RF_P2C	*Bacillus* spp.	AFRI 1_RF_P2C_1	No
3	RF	AFRI 1_RF_P2D	*Citrobacter/Klebsiella/Salmonella* spp.	AFRI 1_RF_P2D_1	No
3	RF	AFRI 1_RF_P3B	*Rhodococcus* spp.	AFRI 1_RF_P3B_1	No
3	RF	AFRI 1_RF_P3E	*Bacillus* spp.	AFRI 1_RF_P3E_1	No
3	RF	AFRI 1_RF_P3F	*Solibacillus* spp.	AFRI 1_RF_P3F_1	No
3	RF	AFRI 1_RF_P4D	*Corynebacterium* spp.	AFRI 1_RF_P4D_1	No
3	RF	AFRI 2_RF_P1B1	*Shouchella* spp.	AFRI 2_RF_P1B1_1	No
3	RF	AFRI 2_RF_P1B2	*Shouchella* spp.	AFRI 2_RF_P1B2_1	No
3	RF	AFRI 2_RF_P1H	*Lysinibacillus* spp.	AFRI 2_RF_P1H_1	No
3	VS	AFRI 2_VS_P1A	*Bacillus* spp.	AFRI 2_VS_P1A_1	No
3	VS	AFRI 2_VS_P1B	*Bacillus* spp.	AFRI 2_VS_P1B_1	No
3	VS	AFRI 2_VS_P1C1	*Shouchella* spp.	AFRI 2_VS_P1C1_1	No
3	VS	AFRI 2_VS_P1C2	*Shouchella* spp.	AFRI 2_VS_P1C2_1	No
3	VS	AFRI 2_VS_P1D	*Bacillus* spp.	AFRI 2_VS_P1D_1	No
3	VS	AFRI 2_VS_P1E	*Caldibacillus* spp.	AFRI 2_VS_P1E_1	No
3	NS	AFRI 2_NS_P1G	*Caldibacillus* spp.	AFRI 2_NS_P1G_1	No
3	FC	AFRI 2_FC_P1B	*Escherichia/Shigella* spp.	AFRI 2_FC_P1B_1	No
3	FC	AFRI 2_FC_P1C	*Bacillus* spp.	AFRI 2_FC_P1C_1	No
3	FC	AFRI 2_FC_P1E	*Robertmurraya* spp.	AFRI 2_FC_P1E_1	No
3	FC	AFRI 2_FC_P1H	*Lysinibacillus* spp.	AFRI 2_FC_P1H_1	No
3	RF	AFRI 3_RF_P1A1	*Paenibacillus* spp.	AFRI 3_RF_P1A1_1	No
3	RF	AFRI 3_RF_P1A2	*Microbacteriaceae* family	AFRI 3_RF_P1A2_1	No
3	RF	AFRI 3_RF_P1E	*Comamonas* spp.	AFRI 3_RF_P1E_1	No
3	RF	AFRI 3_RF_P1F	*Bacillus* spp.	AFRI 3_RF_P1F_1	No
3	RF	AFRI 4_RF_AN_HC_CB_B	*Bacillus* spp.	AFRI 4_RF_AN_HC_CB_B_1	No
3	RF	AFRI 4_RF_AN_HC_CB_C	*Raoultella* spp.	AFRI 4_RF_AN_HC_CB_C_1	No
3	RF	AFRI 4_RF_AN_HC_CB_E	*Corynebacterium* spp.	AFRI 4_RF_AN_HC_CB_E_1	No
3	RF	AFRI 4_RF_AN_HC_WC_A	*Bacillus* spp.	AFRI 4_RF_AN_HC_WC_A_1	No
3	RF	AFRI 4_RF_AN_HC_WC_D	*Bacillus* spp.	AFRI 4_RF_AN_HC_WC_D_1	No
3	RF	AFRI 4_RF_AN_HC_WC_F	*Bacillus* spp.	AFRI 4_RF_AN_HC_WC_F_1	No
3	RF	AFRI 4_RF_AN_HF_CB_A	*Bacillus* spp.	AFRI 4_RF_AN_HF_CB_A_1	No
3	RF	AFRI 4_RF_AN_HF_CB_B	*Streptococcus* spp.	AFRI 4_RF_AN_HF_CB_B_1	No
3	RF	AFRI 4_RF_AN_HF_CB_C	*Bacillus* spp.	AFRI 4_RF_AN_HF_CB_C_1	No
3	RF	AFRI 4_RF_AN_HF_CB_D	*Niallia* spp.	AFRI 4_RF_AN_HF_CB_D_1	No
3	RF	AFRI 4_RF_AN_HF_CB_E	*Bacillus* spp.	AFRI 4_RF_AN_HF_CB_E_1	No
3	RF	AFRI 4_RF_AN_HF_WC_B	*Bacillus* spp.	AFRI 4_RF_AN_HF_WC_B_1	No
3	RF	AFRI 4_RF_AN_HF_WC_D	*Corynebacterium* spp.	AFRI 4_RF_AN_HF_WC_D_1	No
3	RF	AFRI 4_RF_AN_HF_WC_E	*Bacillus* spp.	AFRI 4_RF_AN_HF_WC_E_1	No
3	RF	AFRI 4_RF_AN_HF_WC_F	*Bacillus* spp.	AFRI 4_RF_AN_HF_WC_F_1	No
3	RF	AFRI 4_RF_AR_HC_CB_A	*Bacillus* spp.	AFRI 4_RF_AR_HC_CB_A_1	No
3	RF	AFRI 4_RF_AR_HC_CB_B	*Bacillus* spp.	AFRI 4_RF_AR_HC_CB_B_1	No
3	RF	AFRI 4_RF_AR_HC_CB_D	*Shouchella* spp.	AFRI 4_RF_AR_HC_CB_D_1	No
3	RF	AFRI 4_RF_AR_HC_CB_H	*Bacillus* spp.	AFRI 4_RF_AR_HC_CB_H_1	No
3	RF	AFRI 4_RF_AR_HC_CB_I	*Corynebacterium* spp.	AFRI 4_RF_AR_HC_CB_I_1	No
3	RF	AFRI 4_RF_AR_HC_CB_K	*Bacillus* spp.	AFRI 4_RF_AR_HC_CB_K_1	No
3	RF	AFRI 4_RF_AR_HF_CB_A	*Mammaliicoccus* spp.	AFRI 4_RF_AR_HF_CB_A_1	No
3	RF	AFRI 4_RF_AR_HF_CB_B	*Comamonas* spp.	AFRI 4_RF_AR_HF_CB_B_1	No
3	RF	AFRI 4_RF_AR_HF_CB_C	*Enterococcus* spp.	AFRI 4_RF_AR_HF_CB_C_1	No
3	RF	AFRI 4_RF_AR_HF_CB_F	*Strenotrophomonas* spp.	AFRI 4_RF_AR_HF_CB_F_1	No
3	RF	AFRI 4_RF_AR_HF_CB_I	*Bacillus safensis*	AFRI 4_RF_AR_HF_CB_I_1	No
3	RF	AFRI 4_RF_AR_HF_CB_L	*Robertmurraya siralis*	AFRI 4_RF_AR_HF_CB_L_1	No
4	FC	OCM63_FC_F1C	*Bacillus* spp.	OCM63_FC_F1C_1	No
4	FC	OCM63_FC_F1A	*Bacillus* spp.	OCM63_FC_F1A_1	No
4	FC	OCM63_FC_F1L	*Bacillus* spp.	OCM63_FC_F1L_1	No
4	FC	OCM63_FC_F1Q	*Bacillus* spp.	OCM63_FC_F1Q_1	No
4	FC	OCM63_FC_F1G	*Caldibacillus* spp.	OCM63_FC_F1G_1	No
4	FC	OCM63_FC_F2F	*Heyndrickxia* spp.	OCM63_FC_F2F_1	No
4	FC	OCM63_FC_F1D	*Lysinibacillus* spp.	OCM63_FC_F1D_1	No
4	FC	OCM63_FC_F1O	*Lysinibacillus* spp.	OCM63_FC_F1O_1	No
4	FC	OCM63_FC_F2E	*Niallia* spp.	OCM63_FC_F2E_1	No
4	FC	OCM63_FC_F1E	*Paenibacillus* spp.	OCM63_FC_F1E_1	No
4	FC	OCM63_FC_F1H	*Escherichia/Shigella* spp.	OCM63_FC_F1H_1	No
4	RF	OCM3_RF_MRF_542_CB_A	*Bacillus* spp.	OCM3_RF_MRF_542_CB_A_1	No
4	RF	OCM3_RF_MRF_J25_CB_C	*Acinetobacter* spp.	OCM3_RF_MRF_J25_CB_C_1	No
4	FRF	OCM3_FRF_FRF_10_CB_A	*Paenibacillaceae* family	OCM3_FRF_FRF_10_CB_A_1	No
5	VS	TP1813_CB_A	*Escherichia coli* O171:H25	TP1813_CB_A_EVS_2	Yes
5	RF	TP167_CB_C	*Escherichia coli* O171:H25	TP167_CB_C_ERF_3	Yes

^1^ RF = Ruminal fluid, VS = Vaginal swab, NS = Nasopharyngeal swab, FC = Feces, FRF = Fetal ruminal fluid.

**Table 3:** Distribution of bovine and environmental samples used for phage screening, the number of phages isolated, and the discovery rate per sample type.

Sample Type	Total Number of Samples	Bacteriophages	% Discovery
Ruminal fluid	799	59	7.3%
Feces	30	15	50.0%
Vaginal swab	124	7	5.6%
Fetal ruminal fluid	20	1	5.0%
Nasopharyngeal swab	130	1	0.8%
Drinking water	2	0	0%
Fetal meconium	19	0	0%
Liver abscess	3	0	0%
Liver tissue	10	0	0%
Milk	2	0	0%
Ocular swab	34	0	0%
Runoff water	2	0	0%
Soil	9	0	0%
Soil + feces ^1^	3	0	0%
Tracheal swab	8	0	0%
Uterine swab	15	0	0%
Wastewater	4	0	0%

^1^ Soil + feces samples originated from the soil mixed with bovine feces inside the pens where animals were housed.

**Table 4:** Bacterial host taxonomic classification and number of phages isolated per taxa.

Host Bacterial Taxa	No. Phages	Per. Phages
*Bacillus* spp.	34	41.0%
*Escherichia/Shigella* spp.	8	9.6%
*Shouchella* spp.	5	6.0%
*Corynebacterium* spp.	4	4.8%
*Lysinibacillus* spp.	4	4.8%
*Caldibacillus* spp.	3	3.6%
*Comamonas* spp.	2	2.4%
*Niallia* spp.	2	2.4%
*Paenibacillus* spp.	2	2.4%
*Alkalihalobacillus* spp.	2	2.4%
*Microbacteriaceae family*	2	2.4%
*Robertmurraya* spp.	2	2.4%
*Acinetobacter* spp.	1	1.2%
*Cellulosimicrobium* spp.	1	1.2%
*Citrobacter/Klebsiella/Salmonella* spp.	1	1.2%
*Enterococcus* spp.	1	1.2%
*Heyndrickxia* spp.	1	1.2%
*Mammaliicoccus* spp.	1	1.2%
*Paenibacillaceae family*	1	1.2%
*Raoultella* spp.	1	1.2%
*Rhodococcus* spp.	1	1.2%
*Solibacillus* spp.	1	1.2%
*Staphylococcus* spp.	1	1.2%
*Strenotrophomonas* spp.	1	1.2%
*Streptococcus* spp.	1	1.2%
Total	83	100%

**Table 5:** Sequencing summary and characteristics of 11 bacteriophages from ruminal fluid and vaginal swabs of healthy beef cattle ^1^.

Phage	Sample Type	GenBank Accession Number	SRA Accession Number	Coverage	No. of Reads	Length (bp)	GC Content (%)	Taxonomy
1RFP6A	Ruminal fluid	SAMN35002581	SRR24524243	7872.90	1,217,433	46,700	44.12	*Caudoviricetes* phage
2RFP1A2_1	Ruminal fluid	SAMN35002582	SRR24524242	3640.33	645,720	88,335	38.95	*Felixounavirus* sp.
2RFP1C2_AA	Ruminal fluid	SAMN35002583	SRR24524240	1530.90	416,365	135,443	43.69	*Vequintavirus* sp.
2RFP1E_AA	Ruminal fluid	SAMN35002584	SRR24524239	13976.2	417,407	14,873	44.35	*Caudoviricetes* phage
2RFP1H_AA	Ruminal fluid	SAMN35002585	SRR24524238	1773.77	314,631	88,335	38.94	*Felixounavirus* sp.
2RFP5B2_3	Ruminal fluid	SAMN35002586	SRR24524237	6536.17	541,400	41,250	40.1	*Caudoviricetes* phage
2RFP8A_3	Ruminal fluid	SAMN35002587	SRR24524236	16381.7	665,008	20,216	37.02	*Salasmaviridae* family phage
3RFP5C_2	Ruminal fluid	SAMN35002588	SRR24524235	8174.73	432,243	26,332	41.06	*Herelleviridae* family phage
3RFP5E_6	Ruminal fluid	SAMN35002589	SRR24524234	8794.38	4,325,964	148,554	39.04	*Siophivirus* sp.
TP167CBC_ERF3	Ruminal fluid	SAMN35002591	SRR24524233	3134.37	245,897	39,069	33.94	*Tequatroviru nbeco*
TP1813CBA_EVS2	Vaginal swab	SAMN35002592	SRR24524241	861.104	291,546	168,609	35.41	*Tequatrovirus* sp.

^1^ This table was adapted from our previous genome announcement describing the genome sequences of 11 bovine commensal bacterial isolates (Magossi et al., 2023 [74]).

## Data Availability

All raw genome sequences and assemblies were deposited in the Sequence Read Archive (SRA) and GenBank, respectively, and can be accessed through the accession numbers that are available from our two previous publications describing the genomes and genomic characteristics of the phages and their respective hosts [58,74].
